# Effect of Topical Anaesthetics on Interstitial Colloid Osmotic Pressure in Human Subcutaneous Tissue Sampled by Wick Technique

**DOI:** 10.1371/journal.pone.0031332

**Published:** 2012-02-14

**Authors:** Hans Jørgen Timm Guthe, Torbjørn Nedrebø, Olav Tenstad, Helge Wiig, Ansgar Berg

**Affiliations:** 1 Department of Paediatrics, Haukeland University Hospital, Bergen, Norway; 2 Section of Physiology, Department of Biomedicine, University of Bergen, Bergen, Norway; 3 Hyperbaric Medical Unit, Department of Occupational Medicine, Haukeland University Hospital, Bergen, Norway; 4 Section for Paediatrics, Department of Clinical Medicine University of Bergen, Bergen, Norway; University of Giessen Lung Center, Germany

## Abstract

**Objectives:**

To measure colloid osmotic pressure in interstitial fluid (COP_i_) from human subcutaneous tissue with the modified wick technique in order to determine influence of topical application of anaesthetics, dry vs. wet wick and implantation time on COP_i_.

**Material and Methods:**

In 50 healthy volunteers interstitial fluid (IF) was collected by subcutaneous implantation of multi-filamentous nylon wicks. Study subjects were allocated to two groups; one for comparing COP_i_ obtained from dry and saline soaked wicks, and one for comparing COP_i_ from unanaesthetized skin, and skin after application of a eutectic mixture of local anaesthetic (EMLA®, Astra Zeneca) cream. IF was sampled from the skin of the shoulders, and implantation time was 30, 60, 75, 90 and 120 min. Colloid osmotic pressure was measured with a colloid osmometer. Pain assessment during the procedure was compared for EMLA cream and no topical anaesthesia using a visual analogue scale (VAS) in a subgroup of 10 subjects.

**Results:**

There were no significant differences between COP_i_ obtained from dry compared to wet wicks, except that the values after 75 and 90 min. were somewhat higher for the dry wicks. Topical anaesthesia with EMLA cream did not affect COP_i_ values. COP_i_ decreased from 30 to 75 min. of implantation (23.2±4.4 mmHg to 19.6±2.9 mmHg, p = 0.008) and subsequently tended to increase until 120 min. EMLA cream resulted in significant lower VAS score for the procedure.

**Conclusion:**

COP_i_ from subcutaneous tissue was easily obtained and fluid harvesting was well tolerated when topical anaesthetic was used. The difference in COP_i_ assessed by dry and wet wicks between 75 min. and 90 min. of implantation was in accordance with previous reports. The use of topical analgesia did not influence COP_i_ and topical analgesia may make the wick technique more acceptable for subjects who dislike technical procedures, including children.

**Trial Registration:**

ClinicalTrials.gov NCT01044979

## Introduction

Oedema formation is characterized by accumulation of fluid within the interstitial space and is a result of increased transcapillary fluid flux and/or decreased lymphatic drainage as described for more than 100 years ago by Starling [1] and later quantitatively expressed by the equation [2]: 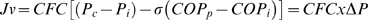
(1)Jv denotes net transcapillary filtration and CFC refers to the capillary filtration coefficient, which is defined as the net filtration in 100 g tissue per minute for each mmHg rise in net capillary filtration pressure. P_c_ and P_i_ are the hydrostatic pressure in the capillaries and interstitial fluid (IF), respectively. COP_p_ and COP_i_ are the colloid osmotic pressures of plasma and IF, and σ is the capillary wall reflection coefficient for plasma proteins. In view of the relations described in the Starling equation, increasing CFC or ▵P can increase fluid flux across the microvascular wall.

The extra vascular forces are smaller than the intravascular pressures, but still important determinants of capillary exchange. Plasma and interstitial colloid osmotic pressure (COP) are therefore essential parameters for understanding fluid exchange during oedema formation and fluid therapy. Sampling of IF by the wick method was first described by Aukland and Fadnes [3] and has been extensively evaluated in animal models [4] [5] [6] [7]. Recently, different groups of patients with fluid disturbances have been studied and have provided important novel data for the understanding and treatment of various disease processes [8] [9] [10]. Some methodological work has been performed in volunteers, with the assumption that similar correlations occur in healthy humans [11] [12].

Implantation of wicks in adults is usually done after intradermal injections of a local anaesthetic, and we know from pilot studies that without local anaesthetic, the wick insertion causes some pain and discomfort. To our knowledge, there are no data regarding COP_i_ in children, mainly because this patient group has not been available for such non-therapeutic research. Minimizing both unpleasant and distressing procedures and trauma from physical and emotional pain is obligate when a paediatric population is involved in clinical research [13]. In order to apply the wick technique for sampling of IF in children it is therefore important to examine how the application of topical anaesthetics on the skin affects the measured values of COP in the IF.

The aim of this study was therefore to evaluate whether topical application of anaesthetics affects measured values of COP_i_. In addition we wanted to examine whether measured COP_i_ is different when IF is obtained from dry or saline soaked wicks and to validate optimal wick implantation time in healthy humans.

## Materials and Methods

### Ethics Statement

The protocol for this study was approved by the local ethics committee (Regional Committee for Medical and Health Research Ethics, Western-Norway) at the Department of Paediatrics, Haukeland University Hospital, where all human experimentation was conducted. Participants were continuously recruited from the clinical staff after simple enquiry. Written informed consent was obtained from all participants involved in the study.

### The wick method

Sampling of IF for determination of COP_i_ and protein distribution were achieved by means of multifilamentous nylon wicks with a diameter of about 0.8 mm (Polyamid no. 8, Norsk Fletteri AS, Bergen, Norway) sewn into subcutaneous tissue. All wicks were sterilized by gamma irradiation (Institute for Energy Technology, Kjeller, Norway). Double threaded wicks were placed on straight sterile suture needles (Acufirm, 210/3, Germany) and tied with a knot at the end. The skin was disinfected with a solution of 0.5% chlorhexidine before inserting the wick in lengths of approximately 5 cm. Except for the knot, ends protruding from the skin were cut of, leaving the knot with 0.5 cm of wick on the outside. Adhesive plastic film (Tegaderm, 3 M Canada Inc.) was placed over both ends of the wick in order to reduce evaporation of fluid. All wicks were placed in parallel, and with a distance of at least 1 cm to each other. At the end of the implantation period, the central part of each wick was rapidly transferred to separate mineral oil filled centrifuge tubes with funnel. Heavily blood stained wicks (judged visually) were discarded and blood stained parts of wicks were cut off if possible. Wicks were centrifuged for 10 minutes at 14.000 rpm and wick fluid could then be collected at the bottom of the tube because the plastic funnel held back the wick during centrifugation. All samples were frozen in plastic tubes (Sarstedt, Reagiergefaße, micro tubes, 1.5 ml) at −20°C until analysis. All procedures were performed under sterile conditions.

Before analysis, room tempered samples were transferred by pipette to a glass capillary tube. The capillary tube was sealed in one end with plasticine (Haematocrit sealing compound, No.74950; BRAND, Germany) and centrifuged in a haematocrit centrifuge (SABA, Haematocrit 20, Hettich) for 3 min. in order to separate mineral oil from wick fluid.

### Colloid osmotic pressure measurements

COP was measured directly with a semi permeable membrane transducer colloid osmometer designed for small fluid samples [14] [15]. The reference camber was filled with isotonic saline (NaCl 9 mg/ml) and a membrane impermeable for molecules greater than a molecular weight of 30-kDalton (PM-30 Amicron, Lexington, MA, USA) was used. Registration of negative hydrostatic pressure, when samples were put on top of the membrane, was measured by a pressure transducer, amplified and recorded (Easy Graph P930, Gould Inc., USA). Before analysing samples the colloid osmometer was calibrated hydrostatically with a saline filled column representing a pressure of 20 mmHg. In order to verify membrane response and to test the accuracy of the whole system, a solution of serum with a known colloid osmotic pressure (COP_c_) was used as a standard reference. After flushing the sample chamber 4 times, 2 µl saline was left for recording zero pressure. Both saline, serum and wick fluid were sucked up by a soft absorbing tissue paper.

### High performance liquid chromatography (HPLC)

In order to determine the distribution of macromolecules in the IF and plasma, a high-resolution size exclusion chromatography using a 4.6 mm (ID)×30 cm TosoHaas Super Sw3000 (Tosoh Biosciences, Stuttgart, Germany) with an optimal separation range for globular proteins of 10–500 kDa was used. One µl of isolated plasma or wick fluid was diluted in 49 µl HPLC buffer to a total volume of 50 µl, and 5 µl of this solution was injected onto the column using an A-905 auto sampler connected to an Ettan 900 LC System (GE Healthcare, formerly Amersham Biosciences). The column was eluted at a constant flow of 0.35 ml/min (P-905 pump) with a 0.1 mM phosphate buffer (pH 6.7) containing 0.1 mM Na_2_SO_4_. Proteins were measured by UV detection at 210 nm using an UV-900 monitor and a 0.7 µl flow cell (path length of 3 mm) connected directly to the column outlet. The HPLC-system was operated by a unicorn software module (ver. 4.12).

### Experimental design

The study was designed as a non-blinded, sequential descriptive study. The study population was enrolled between March 2008 and September 2010.

#### Effect of equilibration time

In order to compare equilibrium time for dry and saline soaked wicks, four dry nylon wicks were inserted subcutaneously into one upper arm and four saline soaked wicks into the contra lateral upper arm in 20 subjects. Thereby, each subject served as his/her own control. Implantation time was 30, 60, 90 and 120 min. on both sides. Ten additional subjects had 3 wicks implanted for 60, 75 and 90 min. respectively.

#### Effect of topical anaesthetics

In order to study a possible effect of topical anaesthetics on the COP_i_ measurements saline soaked wicks were used in 20 subjects. Saline soaked wicks are commonly used in human studies [16] [17] [18] [19] [20] [21] [22] [23] [24] [25] and from our own pilot studies, they cause less pain than dry wicks during insertion. Four wicks were inserted subcutaneously in the same position on each upper arm. On the test arm 2.5 grams EMLA cream was applied and covered with an occlusive dressing for 60 minutes prior to insertion of the wicks (1 gram equals a narrow strip that is 38 mm×5 mm wide containing lidocaine 2.5%/prilocaine 2.5%). Placebo cream was not applied on the control arm before insertion of the wick. During insertion, ten research subjects graded pain on a visual analogue scale (VAS).

A venous blood sample of 5 ml was obtained from all participants after the wicks had been inserted. After the blood had clotted, serum was separated from the sample by centrifugation, 3000 rpm for 10 min. The plasma was immediately frozen in plastic tubes (Sarstedt, Reagiergefaße, micro tubes, 1.5 ml) at −20°C.

#### Statistical analysis

Two tailed paired t-tests were used for comparison of different groups. All values are presented as mean ± one standard deviation (SD), and a p-value<0.05 was considered significant. SigmaPlot 11 (Sy Stat Software/Inc.; Germany) was used for data analyses.

## Results

### Patients

The study population consisted of fifty healthy volunteers, 36 males and 14 females with mean age 36.4±5.9 years (range 29–58) and mean weight of 77.5 kg. There were no complications to wick implantation or blood sampling.

### Wick fluid content and function of colloid osmometer

The average wick length of 5 cm gave nearly 4 µl wick fluid after centrifugation. Two µl was used to measure colloid osmotic pressure, hence duplicate measurements were not possible. Each COP measurement took approximately 4 min. and was performed at laboratory temperatures of 20–22°C. COP_C_ before testing patient IF showed a mean COP_C_ of 27.3±1.5 mmHg (n = 77) with a variance of 2.2.

### Blood contamination of wicks

From a total of 380 inserted wicks, 95 (22%) were discarded because of visible blood. The proportion of blood stained wicks was similar for wicks inserted without and with topical anaesthesia (23% vs. 21%) An additional 9.2% of the wicks had insufficient fluid content for analysis. Blood contamination occurred less frequently in subjects who only had three wicks inserted subcutaneously (13%). Sixty-six% of accepted wicks were classified as light pink, and the proportion of clear wicks was higher in the group receiving topical anaesthesia vs. no anaesthesia (83% vs. 71%).

### Measurement of plasma colloid osmotic pressure

Mean COP_p_ for all subjects was 27.6±1.8 mmHg (range 23.8–31.8).

### Effect of equilibration time

**Figure 1:**
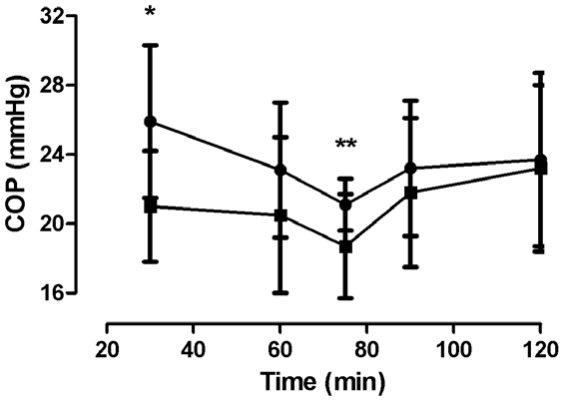
Colloid osmotic pressure and implantation time. Colloid osmotic pressure in interstitial fluid collected from wicks after various times of implantation. Significant difference (p<0.05) between dry (•) and wet (▪) wicks at 30 min. are indicated with (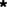) and between dry wicks from 30 to 75 min. with (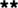).

COP_i_ in interstitial fluid isolated from dry and wet wicks at the different sampling times are shown in Fig. 1. For both dry and wet wicks COP_i_ declined through the first 75 min. after implantation and subsequently increased slightly, but statistically not significantly until all were removed after 120 min. For dry wicks, mean COP_i_ in the same tissue decreased from 25.9±4.4 mmHg after 30 min. of implantation to 21.1±1.5 mmHg after 75 min. (p = 0.043). At 120 min. the mean value was 23.7±5.0 mmHg (p>0.05 from nadir at 75 min.). A similar pattern was seen for wet wicks (21.0±3.2 mmHg at 30 min. vs. 18.7±3.0 mmHg at 75 min. (p>0.05) and 23.2±4.8 mmHg at 120 min (p>0.05) compared to 75 min.) Both dry and wet wicks reached an equilibrium level of 23 mmHg between 75 and 120 min.

### Effect of topical anaesthetics

**Figure 2:**
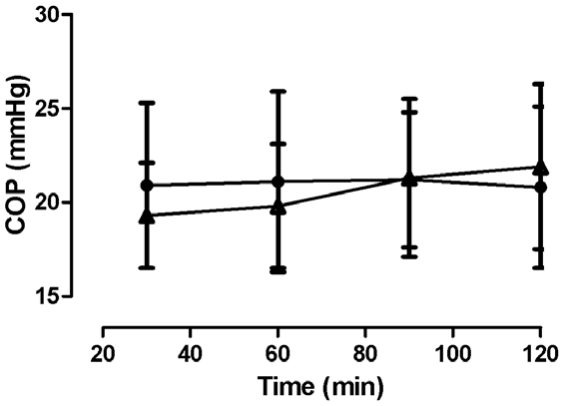
Topical anaesthesia and colloid osmotic pressure. Colloid osmotic pressure in interstitial fluid collected from wet wicks inserted without (•) or after topical anaesthesia with EMLA (▴) plotted against duration of insertion. Mean COP_i_ was 21.0 mmHg in the group without and 20.6 mmHg in the group with EMLA. No significant differences were found between the groups.

There were no significant differences between COP_i_ from wet wicks implanted without or after application of a topical anaesthetic at any of the implantation times (Figure 2). After anaesthesia COP_i_ slowly increased from 19.3±2.8 mmHg at 30 min. to 21.9±4.4 mmHg at 120 min. while the corresponding figures without anaesthesia were 20.9±4.4 mmHg and 20.8±4.3 mmHg (figure 2).

A significant lower VAS score was found comparing wick insertion with EMLA and no topical anaesthesia (4.2 vs. 5.8, p<0.0049) in a subgroup of 10 subjects.

### HPLC of isolated fluid/extravasation of plasma proteins

**Figure 3:**
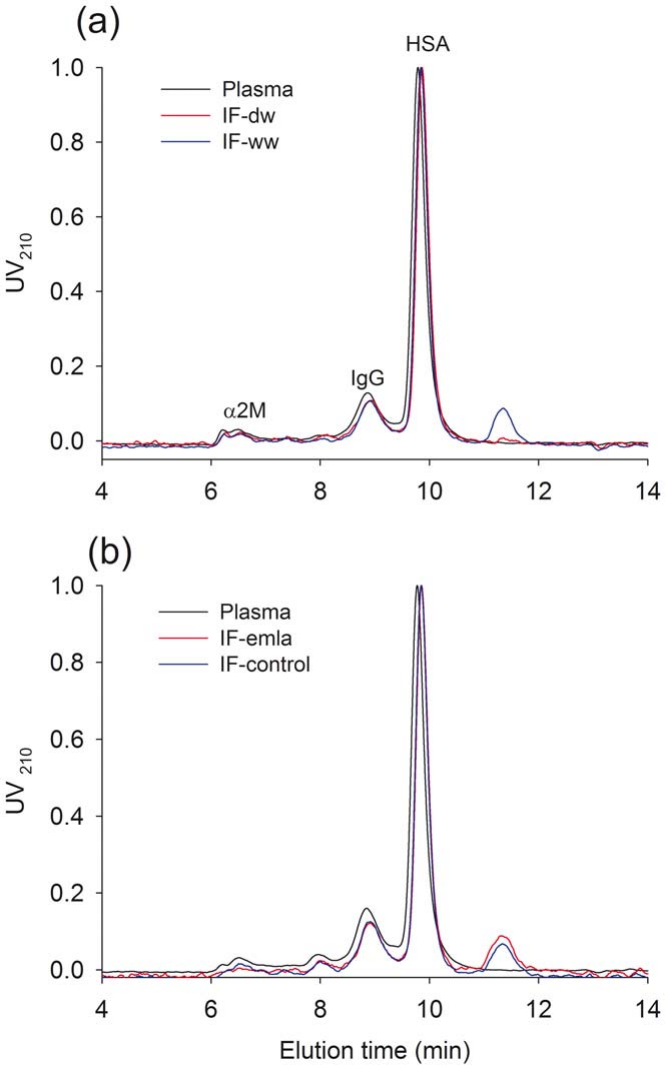
Distribution of macromolecules in interstitial fluid. High resolution size exclusion chromatography of plasma (black line) and interstitial fluid derived from: A; dry (red line) or wet (blue line) wicks, B; wet wicks with (red line) or without (blue line) topical anaesthetics. Wicks were collected after 60 min. of implantation. Ordinate: Absorption units at UV 210 normalized to the highest peak (HSA). Retention time for human serum albumin (HSA), IgG and alpha 2 macroglobulin (α2M) standards indicated. A small haemoglobin peak between 11 and 12 min. retention was usually seen in interstitial fluid.

The size distribution of proteins in fluids isolated from dry and saline soaked wicks and plasma were determined by HPLC. Representative elution patterns are shown in Fig. 3. Protein peaks in plasma corresponded to that of fluids from dry and wet wicks, and the elution pattern was similar in that the globulin peaks were higher than that of albumin. Except for a small haemoglobin peak, which was usually seen in samples from IF, no low molecular weight contaminants were detected.

## Discussion

The transmural colloid osmotic pressure plays major roles in the control of fluid transport across the capillary endothelium and provides important information when fluid is administrated to patients. COP_i_ will significantly influence the transcapillary fluid flux under normal and diseased states [26]. However, the usefulness of measuring COP in clinical practice is still limited due to lack of simple and reliable methods, and discomfort during implantation and removal of wicks. In this study we show that COP_i_ from subcutaneous tissue is easily obtained and that fluid harvesting is well tolerated when topical anaesthetic is used. Most importantly, the use of a topical anaesthetic did not alter the ease of IF harvesting or COP_i_ values when using the wick method for sampling.

To our knowledge, there are few studies of sampling IF without the use of injected local anaesthetics, and the use of topical lidocaine/prilocaine cream prior to wick insertion has not previously been evaluated. In a study by Poulsen et al, one participant had 3 out of 19 wicks inserted without any anaesthesia, with equal results as the mean of all wicks in the same person [27]. EMLA cream, which contains lidocaine and prilocaine, is a low cost topical anaesthetic. It has been found to give equally effective dermal analgesia when assessed with VAS and verbal rating scales (VRS) for venous puncture in children [28] and in adults [29]. The analgesic effect is not affected by skin pigmentation [30]. EMLA cream can be applied from birth (gestational age >37 weeks) and has proven safe and effective in decreasing pain response to venous puncture in neonates in combination with oral sucrose [31]. Intradermal or subcutaneous injection of lidocaine will give almost instant local anaesthesia, but has the disadvantage of an extra uncomfortable skin puncture. In the present study the use of EMLA cream reduced the pain associated with the implantation of wicks as judged from a lower VAS-score. No adverse effects were observed, except that a mild and transient local paleness or redness was observed in some patients. Local injections of different concentrations of lidocaine and prilocaine have shown variable vasoactive responses, although prilocaine has vasodilator properties [32]. Skin reflectance spectroscopy and laser Doppler blood flowmetry show a biphasic skin-blood-flow response with initial vasoconstriction followed by vasodilatation after 3 hours of EMLA cream occlusion [33]. Comparison of vein diameter by Doppler colour ultrasound using EMLA cream or amethocaine indicates a capillary constrictor response rather than venous dilatation [34]. Our results show that despite small variations in skin colour, COP_i_ obtained from wet wicks throughout the implantation time was not significantly affected by the use of EMLA cream indicating that only minor changes in local microcirculation and transudation of fluid occurred across the capillary membrane. Although the manufacturer recommends application of EMLA cream on intact skin for at least 60 min. before procedures [35], and prolongation to 120 min. does not seem to have additional efficacy [30], longer application than 60 min. may contribute to a more pronounced vasoconstriction. Its effect on COP_i_ will presumably be of minor importance since both vasoconstriction and vasodilatation is rapidly reversed after removal of EMLA cream.

Sampling of IF by subcutaneous wicks may cause tissue and vessel damage with subsequent local inflammation and bleeding resulting in increasing capillary permeability to proteins and increased COP_i_. We experienced more frequent bleeding than reported in previous studies [11] [9] where injections of anaesthetic agents were used. One possible explanation may be that a small deposition of liquid can influence local haemostasis with less bleeding as a consequence. Blood stained wicks were discarded in accordance with the recommendations of Aukland & Fadnes [3]. In this study it was reported that a haemoglobin concentration less than 0.2 g/dl in clear and pink wicks [3], indicating a maximum increase in total wick fluid protein content of only 5% as long as the wick was not clearly blood stained. HPLC indicates a low haemoglobin contamination in sampled IF confirming a well operating colloid osmometer. Normal plasma COP in humans is 25–30 mmHg and our findings agree well with this [26].

Subcutaneous implantation of wicks in the upper arm region represents the approximate level of the heart, and variations in body position will only have limited influence on COP_p_ and COP_i_ [12]. All study persons participated in daily activity before and after implanting the wicks. As in previous studies we experienced fluctuations in COP_p_ that were probably due to individual differences within the study population, but were also modestly related to low COP_p_ and low COP_i_. This association was more pronounced after 90 and 120 min. of implantation.

The working mechanism of implanted wicks has been carefully evaluated by Wiig et al where true COP_i_ is best obtained by the crossover method, and optimal implantation time for wicks is believed to be between 90 and 120 minutes in rat models [5]. We found a slow, but statistically non-significant increase in COP_i_ for IF collected from both dry and wet wicks between 75 and 120 min. after insertion which may be ascribed to local inflammation due to mechanical tissue trauma, similar to what has been described in earlier studies on healthy humans [11]. Implantation for longer than 120 min. seems to increase protein concentration in wick fluid from local inflammation with extravasation of plasma proteins [3]. In normally hydrated subcutaneous tissue COP_i_ in wick fluid is found in the range 12.3–18.8 mmHg on the thorax and reduced to 6.5–13.0 below waist level (ankle) [12] [24]. Prolonged horizontal positioning before implantation results in a modest increase in COP_i_. The finding of a higher mean COP_i_ in all our subjects compared to other studies of healthy humans may be due to longer transfer time of wicks from subcutaneous tissue too mineral oil filed tubes. However, this seems unlikely because implantation of wicks was done according to protocol in all subjects as in previous studies in our lab.

The present study has some limitations. In order to optimize evaluation of topical anaesthesia, the subjects as well as the investigator should ideally have been blinded to which arm EMLA cream was applied. Blinding of the investigator would have been possible, but blinding of participants was difficult because EMLA cream is known to create a sensation of numbness. Since there is a tradition of using locally injected anaesthetics in wick studies, future studies should compare injection and topical administration of local anaesthesia before wick insertion.

In conclusion, sampling of IF by wicks in adults was less painful after application of topical EMLA cream than without local anaesthesia. EMLA cream had no significant influence on COP_i_. The COP values obtained in the present study using wick technique were similar to reference data obtained with other sampling methods. Our results indicate an optimal wick implantation time between 75–90 min. Since topical anaesthesia did not affect COP measurements, it is possible to extend the usefulness of the wick method, especially in subjects who dislike technical procedures, including children.
